# Exploring the Impact and Prevention of Epidemics from a New Perspective: COVID-19 Transmission through Express Boxes

**DOI:** 10.3390/ijerph192416884

**Published:** 2022-12-15

**Authors:** Saierdaer Aikebaier, Yinghua Song, Moxiao Li, Jiexin Liu

**Affiliations:** 1 China Emergency Management Research Center, Wuhan University of Technology, Wuhan 430070, China; 2 Hubei Collaborative Innovation Center of Safety Early Warning and Emergency Response Technology, Wuhan 430070, China

**Keywords:** express boxes, pandemic, COVID-19, spatial and geographic transmission, prevention

## Abstract

The number of express boxes worldwide exceeded 170 billion in 2021, and, from several regions in China, tested positive. Therefore, it is important to study the transmission of viruses through express boxes. In this paper, we establish a model of express box virus transmission based on comprehensive consideration of environmental factors, such as temperature, disinfection, humidity, virus release intensity, and volume of vehicle, to study the transmission of express box virus, and explore the spatial and geographic spread variation of express box viruses in China. Several important findings emerged from the study, including: (1) Disinfection can prolong the spread of viruses in the express box for ≥21 h; (2) For every 1 °C rise in temperature, the infected time can be prolonged by ≥1.2 h, and for every 10% rise in relative humidity, the virus transmission time can be prolonged by ≥1.32 h; (3) In an environment suitable for virus transmission, when loaded with 1000, 2000, 4000 express boxes, areas where the express delivery time exceeds 22.56, 18, 14.64 h will face the risk of all the boxes in the carriage being infected. These findings could help public health departments prevent the risk of virus transmission from express boxes.

## 1. Introduction

In the past two years, pandemics such as COVID-19 have had serious negative public health and socioeconomic impacts on countries, and the spread of such infectious diseases has slowed the pace of human social progress. To better prevent such pandemics, research literature on COVID-19 is emerging, and scholars from different countries are studying the impact of COVID-19 from their own perspectives, which will help to address social issues during epidemics in various countries. In the past year, there have been many cases of positive tests for the coronavirus in express boxes or infected courier service workers in several Chinese provinces, and China is currently firmly implementing a dynamic zero prevention and control policy to strictly prevent the spread of the coronavirus. Based on the above, and in order to strengthen the research results in the field of public health, this paper explores virus transmission from a new perspective, conducts research in the field of express box virus transmission, and discusses the corresponding recommendations for disease prevention.

It is known that the virus is affected by the environment and will show different transmission trends under different conditions. In a study of the effect of temperature on COVID-19 transmission in 427 cities worldwide, Wang M found that the cumulative number of confirmed cases peaked at 8.72 °C, each 1 °C increase in mean temperature in low temperature areas was associated with a 0.83 increase in the number of confirmed cases in cities, and the number of urban confirmed cases decreased by 0.86 cases for each 1 °C increase in average temperature in hot areas [1]. A research team from the University of Hong Kong found that coronaviruses can survive for more than 4 days in culture media and that the viruses are highly stable at 5 °C [2]. Other scholars studying the spread of the COVID-19 virus in different temperatures, humidity, and closed or open environments found that cold and dry conditions were enhancers of virus spread, and these factors were further enhanced in closed environments [3,4,5,6,7,8]. Coronaviruses and infectious diseases with similar transmission characteristics require some pathway to cause spatial spread on a global scale. Cui MJ [9] systematically analyzed and compared the differential characteristics of spatial and temporal transmission of SARS and COVID-19 and analyzed the causes by combining the transmission characteristics of the virus itself and factors such as traffic and temperature and found that in addition to the transmission ability of the virus itself, the human transportation system was the main reason affecting the spatial long-distance leap spread of SARS and COVID-19. Thomas M [10] found that a one-scalar-unit increase in weekly bus use was associated with a 1.38-fold increase in the incidence of COVID-19 pneumonia in metropolitan areas of the United States during the pandemic, and a single-scalar-unit increase in weekly rail train use was associated with an increase in incidence 1.54-fold correlation. Many scholars have also constructed models of spatial geographic spread of viruses using shipping aircraft as a virus dispersal pathway, and found by calculation that air transport networks, as a result of modern social development, would increase the risk of spatial spread of infectious diseases while reducing long-distance travel distances [11,12,13]. This provides a reference guide for this paper to study the spatial geographic spread of coronaviruses in different environments.

The study of how COVID-19 is transported by express cartridges leading to spatial spread is most critical to understand the mechanism of coronavirus transmission. Coronavirus is transmitted mainly through droplets or aerosols in close contact with an infected person [14,15]. However, it has been shown that persons can be infected through contact with contaminated objects and surfaces. Further, it was emphasized that contaminated object surfaces play a non-negligible role in pathogen transmission [16,17,18,19]. Kray A found object transmission to be a very important source of transmission risk by studying COVID-19 through viral modeling [20]. When Pastorino B conducted a study on the transmission of the coronavirus, they found that the infectivity of SARS-CoV-2 was significantly maintained in the presence of proteins regardless of the surface type, suggesting that dust mites infected with the virus play a key role in the indirect infection of objects [21]. Ong S studied SARS-CoV-2 virus and found that the virus could complete self-inoculation and multiply on the skin surface after contact with infected objects, suggesting that objects are a greater threat as a means of virus transmission [22]. This provides theoretical support for this paper to study the transmission of viruses in express boxes.

In summary, the study of virus transmission in express boxes, and its spatial and geographic spread by delivery, has a practical and scientific significance. Therefore, in this paper, based on the characteristics of express box transportation, the express box infectious disease model is established based on the comprehensive consideration of environmental influencing factors, such as temperature, disinfection, humidity, virus release intensity, and express vehicle transportation volume. Subsequently, the model calculation results are combined to explore the changes of virus spatial and geographical spread due to express box transportation, with the aim of providing prediction support and theoretical suggestions for virus prevention in public health-related fields, to better cope with future infectious disease pandemics.

## 2. Method: Express Box Dynamic Model

Theoretical knowledge related to infectious disease dynamics is the basis of this study, which is a mathematical model of infectious diseases established by Kermack and McKendrick through mathematical and kinetic knowledge [23]. Infectious disease dynamics models quantitatively reveal the main characteristics of infectious diseases through hypotheses, parameters, variables, and the links between them, rely on data from early outbreaks, incorporate future uncertainties, help discover the mechanisms of infectious disease transmission, and scientifically predict epidemic trends [24]. The SIR model and SEIR model are two classical models in the field of infectious disease dynamics, which classify the population during a pandemic into: susceptible (*S*) latent population (*E*), infected (*I*), and cured/recovered (*R*). Further, susceptible people have the probability *β* to be infected, susceptible people transform into infected people according to the rate *α* (*α* is the reciprocal of the average incubation period), and infected people have the probability *γ* to recover into cured/recovered people. During the pandemic, many scholars have used the above two models to predict the development trend of COVID-19 [25,26,27]. This paper is based on the existence of realistic cases of infected express boxes, combined with the fact that coronaviruses themselves resemble soft colloidal spheres, with the function of adsorbing on the surface of objects and suspending in the air, and can remain highly active on the surface of inanimate objects, transfer and hide [20,28], also relied on the findings that coronaviruses can transfer and spread on the surface of objects, such as leather, for up to 48 h [29], to study the transmission of infection in express boxes caused by the attachment and transfer of coronaviruses on the surface of paper or plastic-type express boxes.

First, in this paper, we establish the express box virus transmission model, Equation (1) (where: *S* is the susceptible box, *I* is the infected box, and the susceptible box has the probability *β* to be infected, and *N* is the total number of boxes in courier transportation). Notably, this model has no cured/recovered (*R*), and combined with the characteristics of virus fomite transmission, there are two reasons: (1) *R* in the original model represents patients who have recovered, and the virus cannot be detected. However, the coronavirus, when attached to a high-touch surface such as an express box, will have a relatively strong activity and transmission capacity to spread to new individuals within tens of hours [30], during which time the virus does not disappear. (2) The incubation period of the coronavirus can be as long as 3–14 days [31], which means that there is a lag in the time when the virus is checked positive, and the incubation time of the virus exceeds the total time assumed by the model. This indicates that the virus has a potential risk of transmission during the entire express box transport cycle.

Secondly, it was learned through a literature review that the activity of indirect transmission of coronaviruses on the surface of objects diminishes with time, and that this change in the possibility of decline depends on the influence of surrounding environmental factors [32]. After the investigation of express box transportation, it was found that the main transport environment of express boxes is closed, and the factors that can have an impact on virus transmission in express box transportation are the following: (1) Disinfection, the preventive measures for the express field after the epidemic are mainly the disinfection of the transport environment before shipment, and disinfection is currently the key link to contain and interrupt the spread of COVID-19 [33]; (2) The volume of the carriage in express box transport, which is a constraint affecting the range of virus transmission and the volume of express box transport; (3) Temperature, which can directly affect the active degree of the virus in express transport, which plays an important role in virus transmission [1,2,3,4,5,6,7]; (4) Virus release intensity, the intensity of free transmission of the virus itself without interference, correlation with the basic regeneration number of virus transmission, which will directly determine the intensity of transmission of different viruses in the initial stage [31]; (5) Humidity, where lower humidity increases the persistence of coronaviruses [7,8]. Additionally, it has been demonstrated that relative humidity is negatively correlated with the global spread of COVID-19, with a 0.19% decrease in the percentage of new cases for every 1 percentage point increase in relative humidity [34]. If indoor relative humidity increases from 46.4% to 66.1%, the number of COVID-19 confirmations decreases by 14% [35]. It is also known that coronaviruses can remain viable on plastic surfaces for up to 5 days in 40~50% humidity environments, and viral viability is rapidly lost at RH > 95% [36]. In order to incorporate the above five factors into the courier box virus transmission model, this paper builds on the existing research on virus transmission trends in closed environments [37]. The environmental influence *ρ*, Equation (2), that affects the activity of express box virus transmission was constructed. The environmental impact value *ρ*, of the express box virus, as a parameter that combines the impact of multiple virus transmission, can help researchers set different environmental conditions in the simulation to achieve the recovery of the real virus transmission environment.

Express box dynamic model:

(1) $$\left\{ \begin{array}{l} \frac{dS}{dt}=-\beta SI\\ \frac{dI}{dt}=\beta SI\\ N=S+I \end{array}\right.$$

Environmental Influence:

(2) $$\rho =\frac{TDoIt}{VH}$$

The factors in Equation (2) have the following characteristics in this study:

*T* = Temperature. In this paper, through the investigation of the express transportation environment, we found that the average temperature in the express carriage is usually kept between is 15~25 °C, there are also special cases, such as in low temperature areas and night driving, the lowest 10 °C, and the highest temperature appears in the summer daytime, the temperature can reach 30 °C. Therefore, the range of values of temperature *T* in this model is set from 10 °C to 30 °C, and the virus propagation activity decreases as the temperature increases.

*D* = Disinfection. Standard disinfection of carriages as well as express cargo at the time of departure, which will determine the base number of viruses in transport, is divided into two cases in this paper, sterilized and unsterilized.

*o* = Virus release intensity. In this study, free propagation without intervention.

*V* = The volume of the carriage of the express box delivery vehicle.

*H* = Relative humidity. This paper draws on the above-mentioned literature to define the range of humidity in different environments and integrating the range of 40–60% of indoor health humidity and the relative humidity in the closed container truck can reach 95%, set the model minimum relative humidity of 35% (the virus has high activity in dry environment), and gradually increase the relative humidity. The virus activity decreases until the virus activity disappears rapidly when the *RH* is 95%.

Finally, according to the knowledge related to infectious disease kinetics, it is known that the infectious rate *ρ* of the infected courier box (*I*) and the susceptible box (*S*) in the vehicle is proportional to both vehicle travel time and transmission density [25], then combined with Equation (2), the express box virus transmission rate can be expressed as Equation (3) (*b* is a constant coefficient proportional to that obtained by fitting the actual data). In combined Equations (1)–(3), can be found the number of individuals in the car infected with express boxes.

(3) $$\beta =b\;\rho \;t=b\;t\frac{TDoIt}{VH}$$

### 2.1. Calculation

#### 2.1.1. The Model Simulation Values Are Set

This study based on the full investigation of the actual express transportation environment, the total time of express transportation was set as 3 days (the average of express transportation in China is <72 h). The viral transmission space was set as the loading volume *V* = 50 m^3^ of medium-sized vehicles of common express vehicles. The primary COVID-19 free dispersal intensity *o* = 0.075. In this study, it was assumed that only one delivery box was attached to a sufficient number of highly viable viruses at the time of departure of the delivery vehicle, *I* (*o*) = 1. The temperature *T*, inside the vehicle was set in the range of 10–30 °C. The base relative humidity *RH* was set at 35% and gradually increased by 20% until it reached 95%. Similar numerical settings were made in the spatial geographic spread study with full consideration of realistic conditions (Table 1). The above settings can be used to obtain the model calculations for the propagation of the express box virus under different conditions.

#### 2.1.2. Effect of Temperature Change on Virus Transmission in Express Boxes

**Figure 1:**
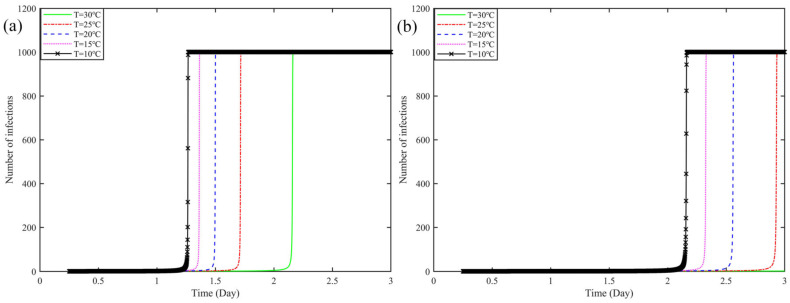
Effect of temperature change on virus transmission on express boxes: ((**a**) Unsterilized, (**b**) Sterilized, and the subfigures show the lines represented by the different temperatures).

Figure 1 shows the effect of temperature change on virus transmission when loading 1000 pieces of express boxes. Figure 1a shows the effect of the change in temperature inside the vehicle without being sterilized, and the effect on the change of virus transmission when *T* was from 30 °C to 10 °C. It can be seen from the figure: With the increase of temperature *T* value from 30 °C to 10 °C, the number of infected boxes at the same time are increasing, from the beginning of *T* = 30 °C to infect 1000 cargoes requires 2.165 days (51.96 h), to *T* = 10 °C, only requires 1.275 days (30.6 h). Compared to the former, shortened 20.36 h. It shows that the number of infected boxes in the car tends to decrease as the temperature increases.

Figure 1b shows that at the time of departure, the car and the box were sterilized, and the effect of the value of the car temperature on the change of virus transmission in the car when it was lowered from 30 °C to 10 °C. If the number of infected boxes ≤ 10 in 3 days, it will not show in the figure. When the above conditions are met, and the *T* from 30 °C to 25 °C, the number of infected express boxes does not exceed 10, in a 3-day period. When the *T* from 25 °C to 10 °C, the number of infected boxes gradually increased, from *T* = 25 °C, when the infection of 1000 pieces requires 2.93 days (70.32 h), to *T* = 10 °C, when the infection of 1000 pieces only requires 2.18 days (52.32 h), shortened 18 h.

After comparing Figure 1a,b, it is clear that disinfection has a greater impact on virus transmission, when the temperature gradually drops from 30 °C to 10 °C. In the absence of disinfection, different temperature conditions infect the entire carriage, taking 30.6 h at the fastest, to 51.96 h at the slowest. With the disinfection, infecting the entire boxes takes 52.32 h at the fastest, and no more than 10 boxes infected in 3 days at the slowest. Further, we can learn from the graph, that the virus transmission rate only starts to rise significantly when the disinfection of the car and express box is done, and the temperature inside the car is below 25 °C.

#### 2.1.3. Effect of Relative Humidity on Virus Transmission

**Figure 2:**
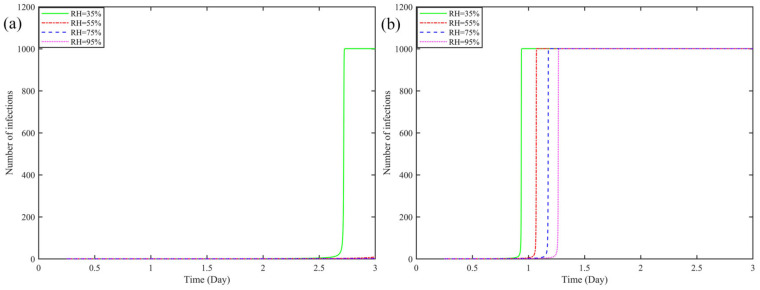
Effect of humidity change on virus transmission in express boxes when (**a**) sterilized and *T* = 30 °C, (**b**) unsterilized and *T* = 10 °C (The subfigures show the lines represented by the different relative humidity).

Figure 2 shows the effect of *RH* change in the vehicle on virus transmission when loaded with 1000 pieces. Figure 2a shows when the vehicle is disinfected and the temperature *T* inside the vehicle is the least suitable for virus transmission during the driving process, when *T* = 30 °C, and changes with the increase of *RH* of ambient relative humidity (*RH* = 55%, 75%, 95%, the number of infected boxes ≤ 10 in 3 days is not shown in the figure). As can be seen from Figure 2b, the overall number of infections showed a linear decline as *RH* increased. In this condition, the model assumes that the entire compartment with 1000 boxes is infected only when the relative humidity *RH* = 35%, it takes 2.73 days (65.52 h) for the entire boxes to be infected, but with *RH* ≥ 55%, the number of infected boxes does not exceed 10 in 3 days.

Figure 2b shows the trend of the effect of increasing *RH* on the risk of virus transmission in the car, when the car is not disinfected and the temperature inside the car is optimal for virus transmission, T = 10 °C. It can be seen from Figure 2b, that as *RH* increases from 35% to 95%, all the boxes are infected, from the fastest need of ≈0.94 days (22.56 h), to the slowest need of ≈1.27 days (30.48 h), the time is reduced by 7.92 h. At *RH* = 35%, it takes 2.73 days (65.52 h) in Figure 2a to 0.94 days (22.56 h) and in Figure 2b, a reduction of 42.96 h.

#### 2.1.4. Effect of Number of Courier Boxes on Virus Transmission

**Figure 3:**
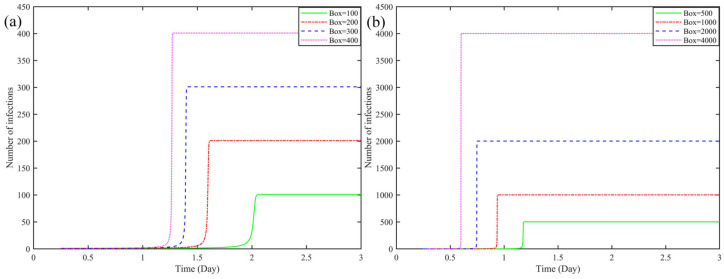
Effect of changes in the number of express boxes on virus transmission in an environment suitable for virus ((**a**,**b**) The subfigures show the lines represented by the different number of boxes).

Figure 3 show the virus transmission time as the boxes increases when the carriage is not disinfected, and when the temperature and humidity inside the carriage are optimal for virus transmission, *T* = 10 °C and *RH* = 35%. As the number of boxes increases, the infected time accelerates, and the amount of boxes and the time are inversely proportional. From 49.44 h for infecting 100 boxes to 14.64 h for infecting 4000 boxes, shortened 34.8 h.

### 2.2. Analysis of Results

The corresponding simulation results can be further summarized through Figure 1, Figure 2 and Figure 3 (Table 2 and Table 3). By analyzing Table 2 and Table 3, and combining them with the model, it can be found that the factors influencing the transmission pattern of COVID-19 in the express box have the following characteristics:

Disinfection. Disinfection directly affects the original number and transmission rate of viruses in the express box, its impact rate on the model ≥ 71%.

Temperature. As express transportation needs to experience day and night, different temperatures have a large impact rate on the virus, in the model the temperature impact rate ≥ 17.5%, per temperature change ± 5 °C.

Relative humidity. Each 20% increase in *RH*, has an effect rate of ≥23.4% on the model.

**Figure 4:**
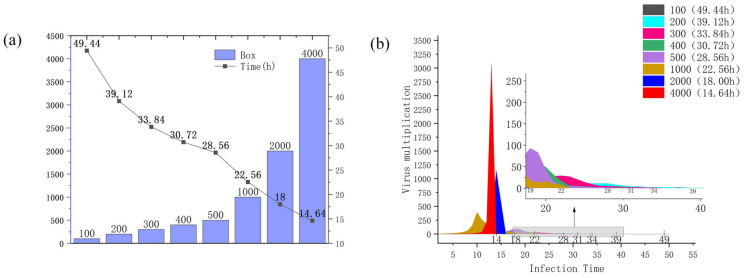
(**a**) Relationship between the number of boxes and the time of infection, (**b**) Change of virus infection rate at different number of boxes.

**Figure 5:**
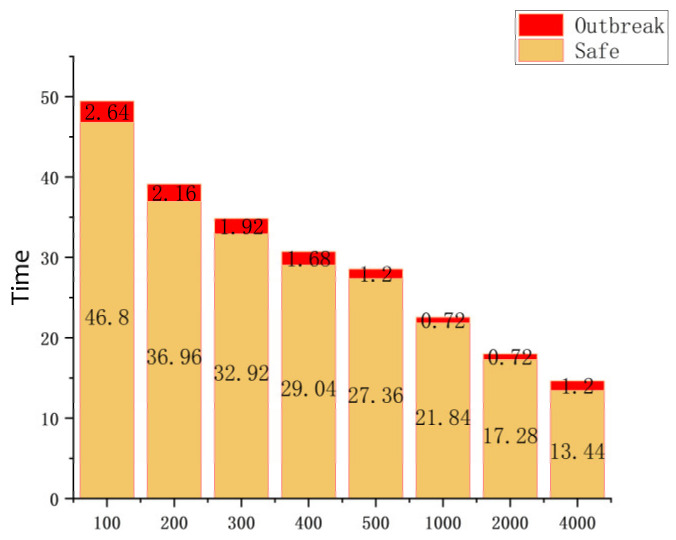
Virus outbreak nodes (virus propagation growth rate ≥ 50%. Red) and the safe time node that can effectively control the virus (virus transmission growth rate < 50%. Yellow).

Boxes. From Figure 3a,b, it can be seen that when the number of express boxes is higher, the faster the infection time of courier in the transport vehicle, and the relationship between its change and the model is shown in Figure 4 and Figure 5. In addition, it can be seen from Figure 4a,b, that as the number of boxes increases, the infection speed is accelerated, and the infection time is rapidly reduced. It takes 49.44 h to infect 100 boxes, but only 14.64 h to infect 4000. Among them, the transmission rate of the COVID-19 virus in boxes is more than 3000 times when the express boxes are 4000, which is nearly more than 2000 times higher than the transmission rate at 1000 boxes. The number of courier boxes is another important variable that affects the whole model.

#### Analysis of the Possibility of Preventing the Express Box Virus

As shown in Figure 4b, we can see that in a very short period, the virus spreads from a smooth growth at the beginning to a sharp increase. Then, if it is possible to know that the virus only started to have a different propagation change at a certain node and set the prevention threshold according to this propagation node, and do the corresponding prevention work, it is of great significance to the research of controlling the spread of the express box virus. Through the calculation of the model, combined with Figure 4b, we found that the number of infections increased exponentially when the virus infection rate was ≥50%, so the point when the virus infection rate reached ≥50% was selected as the critical prevention threshold point for the spread of this express box virus, resulting in Figure 5. Subsequently, this prevention threshold is used as the node, and the time from courier dispatch to this node is called “safe time” (the growth rate of COVID-19 virus transmission in the transport box is ≤50%, and the total transport time is ≥90%), so that we can get the area with the low virus infection number. Finally, according to the proportion of the express boxes in the safe time to the total infection time, combined with the proportion of the boxes outside the safe time (virus outbreak area), we get Table 4.

First, it is known from Figure 5 that the virus rapid outbreak node decreases with the growth of boxes, which is inversely proportional to the cargo volume. It means that the larger the cargo volume is, the shorter the outbreak node time is. Secondly, it can be learned from Table 4 that the ratio of infected cargo volume to total cargo volume after the virus outbreak is ≥90%, the number of infected express boxes in the safe area is small, and the safe area accounts for a long time. This shows that the spread of the express box virus can be effectively controlled by the time advantage.

**Figure 6:**
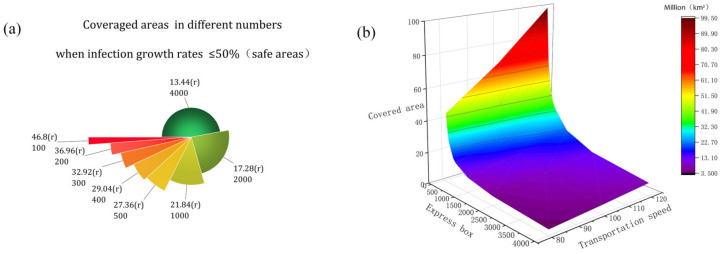
(**a**) is the radius of the optimal geographical area for the prevention of courier box viruses in different number of express boxes and (**b**) is the effect of courier vehicle travel speed and the number of boxes on the optimal area for prevention.

Secondly, based on this safe time, combined with the actual express box transportation, we can infer that the virus in the courier car compartment only starts to break out at the specific range covered by the car in a straight line, from which we can assume: the safe time before the virus outbreak as the radius (r) of the best prevention area in the spatial and geographical spread of the express box virus. Further, it is derived that the safety area for express box virus propagation is shown in Figure 6a. Moreover, we know that vehicles travel different distances at different driving speeds, so in order to better express the relationship between courier transportation speed and the area of safety area it covers, we construct Figure 6b. In this paper, we want to use Figure 6a,b to derive the optimal area of prevention area in courier virus spatial and geographical propagation to improve the accuracy of prevention efforts.

Figure 6a,b shows that the optimal radius (r) for express box virus transmission prevention is shortening as the volume of shipments grows, and with it, the area of the optimal area for prevention and control is shrinking. When there are 100 boxes needing 46.8 (r), to 4000 boxes needing 13.4 (r), the radius difference is 33.4 (r). From Figure 6b, it can be seen that under the same conditions with the acceleration of express transport speed, from 80 km/h to 120 km/h, the best area of prevention and control can be increased by more than double, indicating that improving driving speed in express transport vehicles can effectively increase the best area of prevention and control, reduce the risk of spatial and geographical transmission of the express box virus.

## 3. Spatial and Geographic Spread of the Express Box Virus in China

It is reported that in 2020, China’s courier service companies’ annual business volume was of 83.36 billion pieces, an increase of 31.2% a year, nearly 60 pieces of express boxes per capita, about twice the global average. In 2021, China’s courier companies completed 108.03 billion, an increase of 29.9%. Further, the first confirmed local cases of Omicron were found in Beijing and Shenzhen in the express box cases released by China News in January 2022. Such a large base of courier boxes, combined with the fact that there have been cases of express boxes infected by COVID-19, suggests that the express box sector faces a relatively serious infectious disease prevention challenge.

**Figure 7:**
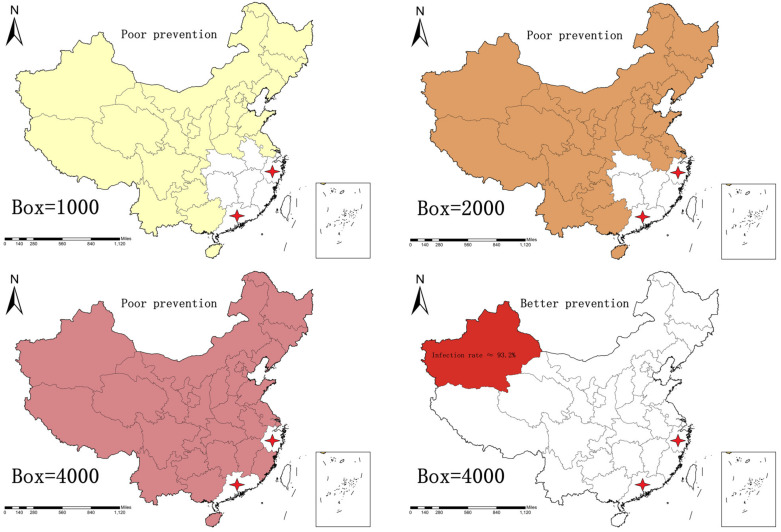
Spatial-geographic influence of the number of express boxes on the transmission of the virus (the first three are the most suitable environment for virus transmission. The last one is the unsuitable environment for virus transmission).

We selected the national courier business volume data of China in 2020 (China Postal Network) and found that the number of courier boxes in Guangzhou and Zhejiang accounted for more than 48% of the courier sending and receiving data in 31 provinces and that using Guangzhou and Zhejiang as references to study the spread of the express box virus within 31 provinces, can effectively reflect the real situation. Combined with part 2.2 of the article, select 1000, 2000, 4000 boxes as the volume of transported goods, with 22.56, 18, 14.64 h as the time point when all the express boxes are infected during transportation, combined with the actual express transport vehicle driving route (the network navigation software as the transport speed and time), assuming that the express transport vehicle from Guangzhou and Hangzhou departure, set the provincial express unit as the destination, vehicle travel average time of 80 km/h. In which, the transport environment is divided into two categories: (1) environment suitable for virus transmission (not disinfected, average temperature of 10 °C, and 35% relative humidity at the time of transport); (2) environment unsuitable for virus transmission (disinfected, average temperature of 30 °C, and 95% relative humidity at the time of transport). Additionally, using the time of arrival of the express box in the province as the time of infection, and marking the color when the province is infected. The spatial and geographic extent of the express box virus infection through transportation is predicted, as shown in Figure 7.

### Preventive Measures for Express Box Virus in Space and Geography

In Figure 7, the first three figures show that the courier box departs from Guangdong and Hangzhou in a suitable environment for virus transmission. When the car is loaded with 1000, 2000, 4000 express boxes, if the delivery time exceeds 22.56, 18, or 14.64 h, then all the delivery boxes in the car will be infected, which means that 74%, 81%, and 94% of China’s provinces will be at high risk from the spread of the express box virus. However, the last graph in Figure 7 shows that in an environment unsuitable for virus transmission, when the car is loaded with 4000 courier boxes, the probability of all the couriers in the car being infected is 93.2% when only one province is receiving this batch of boxes. It is inferred that, according to the trend of express box virus transmission, improving preventive measures for environmental factors of transportation can effectively reduce the risk of virus transmission.

## 4. Discussion

First of all, after the survey and research on the courier field, it was found that the global courier volume showed a gradual upward trend, especially after 2020, when people’s willingness to purchase online increased due to various restrictive precautionary measures for COVID-19 enacted by many countries. Express boxes have the characteristics of large number, frequent contact with humans, and relatively strong cross-regional, which are likely to become an important channel for virus transmission, and there have been reports from countries that have tested positive for COVID-19 in express boxes. Therefore, facing the risk of infectious diseases that may come from courier boxes, this study wants to improve the perception of risk by analyzing the changes in the transmission of viruses from express boxes, and to promote to the health sector to strengthen preparedness and improve response measures [38].

Second, for virus transmission in fomites like express boxes, we need to be clear, while direct transmission is important, the model suggests fomites can also transmit, which is important for exposures that are not in-person. Therefore, fomite transmission may be an important source of risk [20]. Media, temperature, relative humidity, ventilation, and material type have an impact on the persistence of coronaviruses in fomite transmission, and the fact that viruses persist long enough on high contact surfaces suggests that the environment is an important influence on virus transmission [22,30]. In this paper, based on the study of coronavirus transmission in fomites, by establishing environmental impact values *ρ*, we wanted to understand the trend of virus transmission between objects that are frequently touched in human daily life such as express boxes, and further predict the impact of virus transmission in express boxes by combining spatial geographic transmission. However, for modeling studies of virus transmission through fomites and thus infection of humans, we recommend reconsidering the candidacy impact factor because of the change in the primary environment (from a closed to an open environment) and it is possible that the probability of virus infection varies from person to person due to different protective measures for each person (wearing masks and hand disinfection), which requires additional settings on an experimental basis.

The practical significance of this study is that if the COVID-19 virus is found in the express boxes in a certain area, we can track the relevant batches of boxes, combined with the express box virus transmission model, infer the spatial and geographic range of infection by the express box virus, and make targeted prevention to improve the accuracy of virus prevention.

Admittedly, our work suffers from three major limitations: First, the comprehensive tests of environmental influences in this study need to be improved; for example, this paper sets out to study the changes of temperature and disinfection on virus transmission in express boxes with other factors held constant. As we know that the environment is a complex system, this study can only choose a constant value research method for the simulation test. Second, there is a deficiency for the combination of the express box virus model and Chinese spatial geography; this study sets the express box departure to the provincial point as the total transportation distance, and the infection of the express box in the carriage when it arrives at the province represents the infection of the whole province, and the study of the change of the number of express boxes in the carriage after the express is redistributed through the provincial transfer station needs to be further explored. Third, this paper takes values based on the initial COVID-19 virus release intensity, while for the continuously mutating virus, the virus release intensity keeps increasing, which will increase the risk of virus transmission and spread, which will require changes in future studies.

## 5. Conclusions

The main contribution of this study is to explore the spread of COVID-19 in express boxes and derive the trend variation of virus transmission among boxes, and then combine the virus transmission model with Chinese reality to explore the impact and prevention of the express box virus in spatial and geographical spread. Overall, the environmental factors of express box virus transmission are within the controllable range and focusing on the prevention of environmental factors in the carriage during express box transportation can effectively reduce the transmission of the express box virus. For example, courier transport companies can install ventilation equipment in vehicles and maintain a better environmental level in express box storage compartments by monitoring temperature and humidity measuring instruments, which not only helps in the prevention of infectious diseases, but also helps in the protection of products. In addition, it is important to disinfect the environment at the time of shipment, and if long-distance distribution is required, the risk of virus transmission can be reduced by increasing the frequency of disinfection.

For individuals, when we sign for a delivery, we touch the delivery box packaging, and then we may face a certain degree of indirect fomite transmission, The best personal prevention method is to dispose of the express box packaging after we sign, do not carry it or store it at home, and after signing, we must disinfect the touching parts—hands.

## Figures and Tables

**Table 1:** List of important parameters used in the numerical simulations.

Parameters	Definition	Value Range	References
*T*	Temperature in the carriage in express delivery	10~30 °C	[1,2,3,4,5,6,7]
*H*	Relative humidity in the carriage in express delivery	35~95%	[34,35,36]
*D*	Disinfection of the carriage environment in express delivery	Disinfected or not	[33]
*o*	Free transmission strength of virus in closed environment	0.075	[3,25,31,37]
*V*	The volume of the express box storage environment in delivery	50 m^3^	Volume of conventional box-type express delivery vehicles
Express box	Usually paper material, three-dimensional packaging of the express box	100 to 4000 boxes	Conventional courier vehicle loading capacity
Speed	Speed of express delivery vehicles	80 to 120 km/h	China’s highway speed setting for vehicles
Distribution Route	Express delivery route	The shortest driving route between the two provinces	Navigation Software

**Table 2:** Effect of temperature and disinfection on virus transmission in express boxes (If the number of infections is <10, it is denoted by ‘-’).

Disinfection	Temperature	Relative Humidity	Total Infection Time (h)
No	30 °C	95%	51.96
No	25 °C	95%	41.28
No	20 °C	95%	36.24
No	15 °C	95%	32.88
No	10 °C	95%	30.6
Yes	30 °C	95%	-
Yes	25 °C	95%	70.32 (29.04 Increased)
Yes	20 °C	95%	61.68 (25.44 Increased)
Yes	15 °C	95%	56.40 (23.52 Increased)
Yes	10 °C	95%	52.32 (21.72 Increased)

**Table 3:** Effect of relative humidity variation on virus transmission in express boxes (If the number of infections is <10, it is denoted by ‘-’).

Disinfection	Temperature	Relative Humidity	Total Infection Time (h)
No	10 °C	35%	22.56
No	10 °C	55%	26.16
No	10 °C	75%	28.32
No	10 °C	95%	30.48
Yes	30 °C	35%	65.52 (42.96 Increased)
Yes	30 °C	55%	-
Yes	30 °C	75%	-
Yes	30 °C	95%	-

**Table 4:** Proportion of cargo volume in different boxes to total cargo volume before and after the virus outbreak.

Percentage/Boxes	100	200	300	400	500	1000	2000	4000
Safe area	11%	5%	4.20%	3.80%	3.20%	1.30%	0.50%	0.18%
Outbreak area	89%	95%	95.80%	96.20%	96.80%	98.70%	99.50%	99.82%

## Data Availability

The data presented in this study are available on request from the corresponding author.
